# Scalable photonic-based nulling interferometry with the dispersed multi-baseline GLINT instrument

**DOI:** 10.1038/s41467-021-22769-x

**Published:** 2021-04-29

**Authors:** Marc-Antoine Martinod, Barnaby Norris, Peter Tuthill, Tiphaine Lagadec, Nemanja Jovanovic, Nick Cvetojevic, Simon Gross, Alexander Arriola, Thomas Gretzinger, Michael J. Withford, Olivier Guyon, Julien Lozi, Sébastien Vievard, Vincent Deo, Jon S. Lawrence, Sergio Leon-Saval

**Affiliations:** 1 Sydney Institute for Astronomy, School of Physics, The University of Sydney, Sydney, NSW Australia; 2 Sydney Astrophotonic Instrumentation Laboratories, Physics Road, University of Sydney, Sydney, NSW Australia; 3 AAO-USyd, School of Physics, University of Sydney, Sydney, NSW Australia; 4 Research School of Astronomy and Astrophysics, Australian National University, Canberra, ACT Australia; 5 California Institute of Technology, Pasadena, CA USA; 6 Laboratoire Lagrange, Observatoire de la Côte d’Azur, Université Côte d’Azur, Nice, France; 7 MQ Photonics Research Centre, Macquarie University, Sydney, Australia; 8 Subaru Telescope, National Astronomical Observatory of Japan, National Institutes of Natural Sciences, Hilo, HI USA; 9 Steward Observatory, University of Arizona, Tucson, AZ USA; 10 Astrobiology Center, National Institutes of Natural Sciences, Mitaka, Tokyo Japan; 11 James C. Wyant College of Optical Sciences, University of Arizona, Tucson, AZ USA; 12 Australian Astronomical Optics - Macquarie, Macquarie University, Sydney, NSW Australia; 13 Institute of Photonics and Optical Science, School of Physics, University of Sydney, Sydney, NSW Australia

**Keywords:** Astronomical instrumentation, Integrated optics

## Abstract

Characterisation of exoplanets is key to understanding their formation, composition and potential for life. Nulling interferometry, combined with extreme adaptive optics, is among the most promising techniques to advance this goal. We present an integrated-optic nuller whose design is directly scalable to future science-ready interferometric nullers: the Guided-Light Interferometric Nulling Technology, deployed at the Subaru Telescope. It combines four beams and delivers spatial and spectral information. We demonstrate the capability of the instrument, achieving a null depth better than 10^−3^ with a precision of 10^−4^ for all baselines, in laboratory conditions with simulated seeing applied. On sky, the instrument delivered angular diameter measurements of stars that were 2.5 times smaller than the diffraction limit of the telescope. These successes pave the way for future design enhancements: scaling to more baselines, improved photonic component and handling low-order atmospheric aberration within the instrument, all of which will contribute to enhance sensitivity and precision.

## Abstract (web summary)

Nulling interferometry is a technique combining lights from different telescopes or apertures to observe weak sources nearby bright ones. The authors report the first nulling interferometer implemented in a photonic chip doing spectrally dispersed nulling on several baselines, simultaneously.

## Introduction

With more than 4300 exoplanets confirmed so far^1^, questions of the characterisation of their chemistry and physical conditions have become pressing. Indirect detection methods, such as transit or precision radial velocity observations, have revealed the ubiquity and the diversity of exoplanetary systems. However, few Earth-analogue exoplanets orbiting in the habitable zone of their host star have been discovered and present challenges for either technique. Direct imaging is one of the methods under development to study such systems, not only able to provide orbital parameters but also characterise the surface, the atmosphere, the weather and even probe for signatures of life^2–4^. However, many observational challenges remain.

In order to observe and study planets located in the habitable zone, it is critical to suppress the overwhelming glare from direct starlight over scales of angular resolution much smaller than one arcsecond^5^. The contrast ratio between the faint speck of planetary light and the star ranges between ~10^−4^ for massive, self-luminous hot exoplanets observed in the mid-infrared^6^, to 10^−10^ for Earth-like exoplanets imaged in reflected light from their host star^7^. An exoplanet in the habitable zone (~1 AU) around a star in a nearby star-forming region (~100 pc) has an angular separation from its host star of 10 milliarcseconds (mas hereafter). Such systems lie somewhat beyond the diffraction limit of 8–10 m class telescopes (40 mas at 1.55 microns on an 8-m aperture and 32 mas on a 10-m aperture), and within range of next-generation large apertures. However, atmospheric turbulence blurs the images and limits the uncorrected resolving power to about one arcsecond, hence the need for adaptive optics systems to reach the diffraction limit of the telescope.

Coronagraphy, combined with adaptive optics, is the most developed method to address the needs for simultaneous high contrast ratio at high angular resolution. Several major instruments worldwide such as SPHERE^8^, GPI^9^, SCExAO^10^ and the vortex coronagraphs of Keck^11^ all deploy various forms of coronagraphs. One performance metric for such devices is the inner-working angle (IWA) conventionally defined as the spatial separation from the optical axis over which off-axis objects would have a 50% throughput of the peak throughput of the coronagraph. Current generation coronagraphs struggle to reach IWAs close to the formal diffraction limit: $\frac{\lambda }{D}$ (where *λ* is the wavelength and *D* the diameter of the telescope)^12^. For a modern large (~8 m) telescope operating in the near-infrared, this limit rules out exploration of habitable zone orbits of Sun-like (or cooler) stars for all but a tiny handful of very nearby systems.

An alternative method is nulling interferometry^13^. Drawing upon long baseline interferometry (or aperture masking), beams of starlight from separate telescopes (or sub-apertures) are brought together where they interfere. The distinction is that a nulling interferometer is configured with a *π* radian phase shift between the beams so that on-axis light will destructively interfere. Consequently, the starlight is cancelled out at the centre of the image plane and often redirected into a separate path. The light coming from an off-axis source carries a phase-shift imposed by the non-axial angle of incidence so that the condition for destructive interference is not met and the light is not nulled. Such an arrangement means that light coming from a nearby companion, such as a planet, may be isolated from the star. Unlike coronagraphs, the effective IWA of nulling interferometers (shortened to “nullers”) depends on the baseline *B* separating the apertures so that $IWA=\frac{\lambda }{2B}$^14^. Also unlike coronagraphs, this effective IWA is not a hard limit; rather (as demonstrated in this paper) spatial structure can continue to be resolved much closer to the star, at the expense of a lower achievable contrast. The primary observable is called the source null *depth*, defined as the ratio of the intensity of destructive over constructive interference, which quantifies the degree of suppression of the light due to spatial brightness distribution of the source. It is recovered by the processing of the *measured* null depth, but biased by the instrumental response and seeing-induced effects. The precision of its measurement is affected by fluctuations arising from various sources, including atmospheric seeing, vibrations, detector noise, etc. This precision can be improved using statistical data-analysis and fitting methods such as described here.

Several conceptual improvements for space-based observatories have been proposed for imaging^15^ and high angular resolution capabilities^16^. Theoretical studies into instrumental effects on the nulled light such as spectral dispersion and polarisation have also been made^17^. Several ground-based nullers have been built, including the BracewelL Infrared Nulling Cryostat^18^, the Keck Interferometric Nuller (KIN)^19^, the Palomar Fiber Nuller (PFN)^20^ and the Large Binocular Telescope Interferometer Nuller (LBTI)^21^. In addition to their technological innovation, these instruments produced a range of important scientific results. For example, KIN, which combined both 10 m Keck Telescopes, performed a major survey of exozodiacal dust around 47 nearby stars, using 10 spectral channels ranging from 8 μm to 13 μm wavelengths (N band)^22^. More recently, the LBTI has investigated the presence of dust closer to the habitable zone of nearby stars as part of the HOSTS survey^23^, also in N band, critical for future exo-Earth imaging endeavours. While these instruments relied on conventional bulk-optical components, the Palomar Fiber Nuller used the spatial filtering properties of optical fibres to improve the instrumental null depth, combining light from two sub-apertures of the 5.1 m Palomar Telescope at K_*s*_ band (~2.2 μm). It also introduced the numerical self-calibration method (NSC)^24^, i.e., the analysis of the statistical fluctuations of the measured null depth to deduce the underlying source null depth, yielding sufficient precision to detect a faint companion in a binary system^25^ and a component of the disk around *AB Aur*^26^.

We have built a nulling interferometer, built upon the GLINT (Guided-Light Interferometric Nulling Technology) framework. Unlike the aforementioned instruments, it performs all operations within a photonic chip. An earlier prototype, (“GLINT South”) deployed at the Anglo-Australian Telescope in Australia, emphasised study of the performance of the photonic technology itself (refs. ^27,28^ and Lagadec et al., manuscript in prep.) under seeing-limited conditions. The design of previous photonic nullers (like PFN) fundamentally limited the nulling to only be performed on a single baseline. The GLINT instrument is optimised for science by taking advantage of wavefront correction provided by the adaptive optics system (AO)^29^ and by being the first photonic instrument capable of nulling multiple baselines simultaneously, with the delivery of six non-redundant interferometric baselines. Its optical circuitry yields dedicated outputs for the destructive interference, the constructive interference as well as a simultaneous photometric monitor for each beam, all reformatted to deliver spectral information by cross-dispersion in a spectrograph. GLINT is integrated into the Subaru Coronagraphic Extreme Adaptive Optics system (SCExAO)^10,30,31^, at the Subaru Telescope.

This use of integrated-optics technology yields a straightforward and scalable approach to combine several apertures—up to spanning the whole pupil for the case of a single large telescope aperture—delivering significant improvement to sensitivity and imaging capability. The other assets of this technology are spatial filtering, flexibility of design, and straightforward implementation of complex optical processing such as GLINT’s spectroscopic back-end. We therefore incorporate all the main characteristics of previous-generation nullers, while adding the features enabled by integrated-optics (most notably multiple, simultaneously nulled baselines), in a single device.

GLINT aims to demonstrate the design of a highly capable, scalable instrument for future large telescopes, such as the Extremely Large Telescope (ELT) or Thirty-Metre Telescope (TMT), and for long baseline interferometers like the Very Large Telescope Interferometer (VLTI). Furthermore, the use of photonic chips facilitates the realisation of advanced architectures such as the multi-tier combiner^16^ or kernel-nulling^32,33^ aiming for robust performance against time-varying instrumental phase.

In this work, we show that the combination of integrated-optics technology with more advanced data analysis algorithms (built upon the numerical self-calibration method) improves the precision of the nulling data, with enhanced performance against both random and systematic errors. The achieved null depth is better than 10^−3^ with a precision of 10^−4^ for all baselines, with *λ*/8 RMS simulated seeing applied. On-sky commissioning demonstrates the performance of GLINT in real condition with the successful determination of the angular diameters of two stars that are 2.5 times smaller than the diffraction limit of the telescope.

## Results

### Description of the instrument

**Fig. 1:**
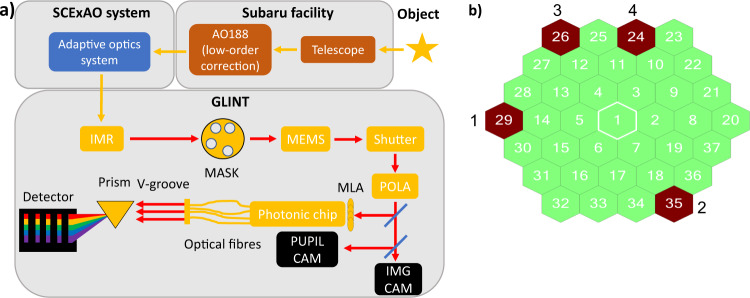
Global schematic of GLINT and configuration of the apertures of the mask. **a** Global schematic of GLINT and its integration within the Subaru Telescope and SCExAO. Within GLINT itself the sequence of optical systems encountered consists of the image rotator (IMR), a pupil mask, a steerable segmented mirror (MEMS), a shutter and a linear polarising beamsplitter (POLA). The science beam is then injected by a microlens array (MLA) into the photonic chip while the dichroic beamsplitter delivers light to cameras viewing both the image plane and pupil plane. The processed beams coming from the photonic chip are spectrally dispersed in the GLINT spectrograph then imaged on the science detector (C-Red 2). **b** Diagram of the segmented MEMS mirror with highlighted segments in red matching the pattern of holes in the mask. Segments 29, 35, 26 and 24 are identified with beams 1, 2, 3 and 4, respectively. The lengths of baselines range from 2.15 m (combination of the beams 3 and 4) to 6.45 m (combination of the beams 2 and 3).

GLINT’s underlying optical design has been described in a previous article^29^ and for convenience is briefly set out here. The Subaru Telescope’s 8-m mirror collects light and firstly routes it to the AO-188 adaptive optics facility^34^ for low-order wavefront correction. Starlight next traverses the SCExAO extreme AO system where it receives high order correction. The beam is finally redirected toward the GLINT optical table (Fig. 1a) with the first element being an image rotator to control the angle of projection of the baselines onto the sky plane. The telescope pupil is re-imaged onto an opaque carbon fibre mask, containing four apertures that provide six non-redundant baselines (Fig. 1b). The pupil is undersized to a maximum baseline of 6.45 m with respect to the size of the primary mirror because of optical constraints. The 8 m-pupil will be filled with more apertures in a future upgrade of GLINT. The mask holes are aligned with those of a corresponding MEMS-based segmented deformable mirror by translating the mask in the pupil plane. Each segment can be individually moved in tip and tilt to maximise the injection of the light into the waveguides in the chip, and in piston to scan the phase of the interferometer arms hence the fringes and adjust the position of the dark fringe. Next, a polariser selects a single linear polarisation state and a dichroic beamsplitter divides the beams between the science channel and alignment-camera channel. Transmitted beams are injected into the photonic chip by way of a microlens array. The beam reflected by the beamsplitter is sent to a two-camera system used for metrology and alignment that is capable of viewing both the image and pupil planes. The image-viewing camera allows setting the point-spread-function (PSF) position such that the beam is aligned with the optical axis of the chip. The pupil-viewing camera is used to align the MEMS mirror segments with the holes in the mask as well as with SCExAO’s telescope pupil mask (which has spiders), all of which appear superimposed. Taken together, these viewing systems enable alignment and monitoring of GLINT’s internal optics, together with registration with respect to SCExAO and Subaru.

**Fig. 2:**
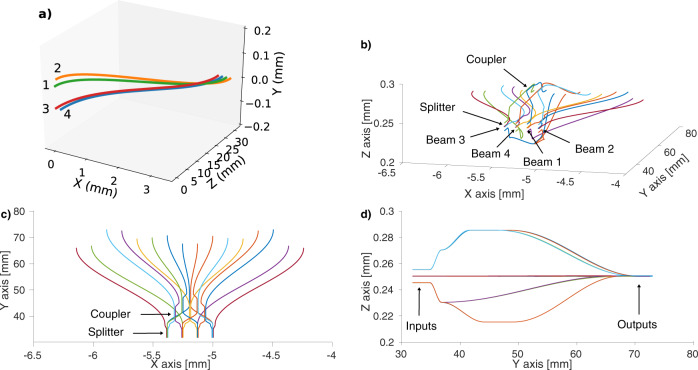
Schematics of the integrated-optics chip. **a** Schematics of the pupil remapper of the chip, coherently transforming the 2D configuration of the inputs (on the left) matching the desired pupil sampling pattern into a 1D configuration (on the right). The waveguide paths have been optimised to match their optical path lengths despite their different routes. The green waveguide is associated with beam 1, orange with beam 2, red with beam 3 and blue with beam 4. **b** Perspective view of the beam combiner of the chip. **c** Plan view in which light propagates from the 4 inputs at the bottom towards the top, encountering 4-way splitters and codirectional couplers. **d** Right-side view of the chip showing the locations of the inputs and the outputs. The inputs, outputs, splitters and couplers are indicated on the (**b**–**d**) diagrams. The axis scale proportions in all the schematics differ for clarity in the drawing.

The core element of GLINT is the integrated-photonic chip (for more detailed technical information, see ‘Specifications of the instrument’ in “Methods”) which performs all the primary interferometric operations on the starlight. The chip consists of two parts. The first one is a pupil remapper (Fig. 2a) which has four inputs, each fed by a sub-aperture of the primary mirror. It coherently reformats the 2-dimensional array of the beams into a one-dimensional configuration suitable for feeding the second part. This is the beam combiner section (Fig. 2b–d) which interferometrically combines each pair of beams and provides monitoring of the injection efficiency. The chip delivers 16 output beams: four photometric taps, six outputs carrying the destructive interference of all six pairs of beams (baselines), identified as null outputs, and six with the constructive interference from the same pairs, identified as anti-null outputs. These outputs are individually redirected into the GLINT spectrograph, located in a stable place elsewhere on the Nasmyth platform via 16 separate optical fibres inserted into a single protected cable. Finally, the spectrally dispersed beams are projected on a C-Red2 InGaAs camera^35^ with <30 e^−^ read noise.

### Characterisation of the photonic chip

The characterisation of the chip informs the parameters required for data processing and the required configuration of the instrument for a night of observation. Therefore, the optical propagation lengths of the waveguides and the characteristics of the splitters and couplers were studied.

The first phase of characterisation included the measurement of optical path length differences between all pairs of waveguides. It was performed by scanning the phase of each baseline (by incrementally pistoning the associated MEMS segment) while the null-channel output intensities (integrated over a 200 nm bandwidth) were recorded. The position of the centre of the fringe envelope was then recovered by fitting with a simple model (a more comprehensive multi-wavelength model is detailed in the subsection “Data processing”). The intensity *I* as a function of delay *δ* is given by:

1 $$I(\delta )=A+B\sin \left(\frac{2\pi }{{\lambda }_{0}}(\delta -{\delta }_{0})+\phi \right){\rm{sinc}}\left(\frac{\delta -{\delta }_{0}}{{\lambda }_{0}^{2}}{{\Delta }}\lambda \right).$$

where *A* and *B* are respectively the incoherent term and the amplitude of the fringe pattern, *λ*_0_ is the wavelength at which the scan is performed and Δ*λ* the bandwidth, *δ*_0_ is the parameter of interest: the optical path difference (OPD) between the two beams at the centre of the envelope. *λ*_0_ and Δ*λ* were known and the other parameters were fitted to the data.

**Fig. 3:**
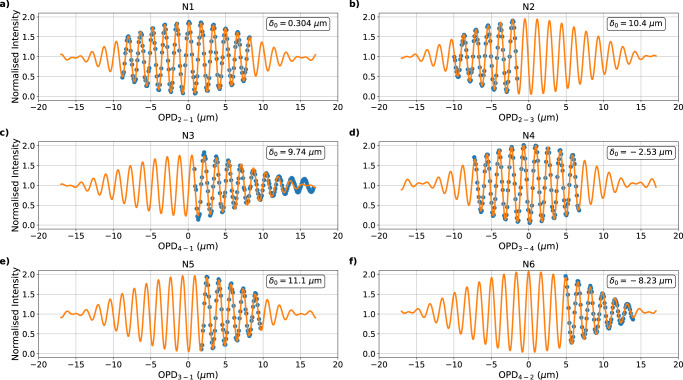
Scanned fringe envelopes for each of the six baselines. Subplots (**a** to **f**) respectively represent the baselines N1 to N6 scanned over the full available range of the optical path difference (OPD) from data taken in July 2019 with illumination from the supercontinuum source within SCExAO. The blue dots are the data and the orange curve is the fitted model. The label of the horizontal axis “OPD_*x*−*y*_” shows the segment of the beam *x* was scanned with respect to the reference beam *y*. The value in each plot is the fitted *δ*_0_.

It is seen on Fig. 3 that, for this optical alignment, only two baselines, N1 and N4, exhibit data that span the centre of the fringe envelopes. To reach the central null the other baselines require an OPD offset that exceeds the mechanical piston range of the MEMS segmented mirror. Furthermore, the optical path lengths are different between all waveguides. Although some level of mismatch is inevitable, it is important to identify and subsequently improve the on-chip cophasing in future chip fabrication rounds.

The most likely cause for the inconsistent optical delays is physical differences in the optical paths through the system for the 4 input beams, either internal or external to the chip itself. Within the remapper or the coupler sections of the chip, non-uniformity in the waveguide engraving process (for example, from small variations in intensity of the writing-laser) would manifest as refractive index variations along the waveguide. Alternatively (or possibly in addition to), mismatched optical paths external to the chip might arise in the alignment of the microlens array with respect to the chip. As will be described, the effects of the OPD mismatch for 4 baselines is presently managed by accounting for the instrumental loss of coherence in the data analysis model, and performing measurements on some baselines sequentially. Improvements in the ULI manufacturing process, including more comprehensive parameter scans and real-time monitoring of waveguide OPD during fabrication, will address these issues in future chip iterations.

The second phase of characterisation focused on the coupling coefficients of the codirectional couplers and the splitting coefficients of the splitters within the chip. The recovered null depth depends on both the coherence of the light and on any variations in flux between the incoming beams. The latter may arise as rapidly fluctuating phenomena (e.g., scintillation and variations of injection) or on longer timescales (e.g., instrumental transmission along differing paths). Measurement of the instantaneous fluxes of all input beams, simultaneous with the interference measurements, avoids inaccuracy arising due to time-varying fluxes (e.g. from fast wavefront errors coupling to injection losses). This represents a more robust solution for flux fluctuations compared to non-simultaneous beam-chopping based methods implemented by previous generation nulling interferometers^19–21^.

**Fig. 4:**
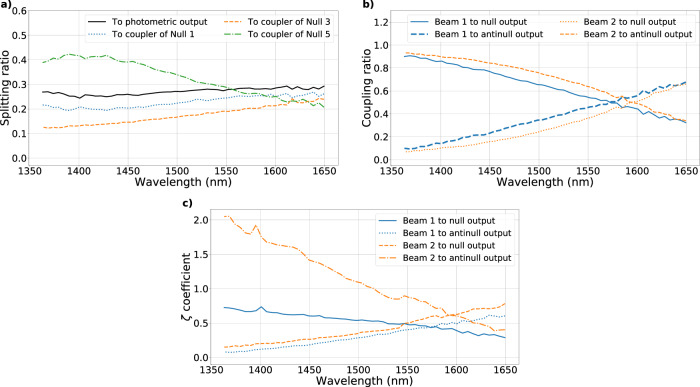
Splitting, coupling ratios and *ζ* coefficients of the beam combiner. **a** Splitting ratios of beam 1 between the photometric output and the three couplers with respect to wavelength. **b** Coupling ratios of the coupler combining the beams 1 and 2 with respect to wavelength. **c** *ζ* coefficients for beams 1 and 2 for the outputs of Null 1 with respect to wavelength.

Detailed knowledge of all splitting and coupling ratios delivered by devices inscribed within the chip was needed to perform an unbiased correction of the photometric contribution to the null depth. The coefficients of the four splitters and the six couplers of the chip were measured as a function of wavelength using the supercontinuum source of the SCExAO bench. The splitting coefficient is defined by

2 $${S}_{x}=\frac{{I}_{x}}{{I}_{p}+{I}_{a}+{I}_{b}+{I}_{c}},$$

where *I* is the flux of the output of interest *x* of the splitter among *p*, *a*, *b*, *c*, which are respectively the photometric tap and the three couplers. Figure 4a presents the splitting coefficients for beam 1 into its 4 components, which are seen to be chromatic over the full waveband. These plots are representative of all splitters, which produce similar results.

The coupling coefficient *C*_*n*_ is defined as the fraction of light in a coupler going to the null output

3 $${C}_{{\rm{n}}}=\frac{{I}_{{\rm{n}}}}{{I}_{{\rm{n}}}+{I}_{{\rm{an}}}}$$

where *I*_n_ and *I*_an_ are the fluxes in the null and anti-null output, respectively. Similarly to the case for the splitters, Fig. 4b shows that the coefficients of the coupler combining the beams 1 and 2 are a function of wavelength; again a finding representative of all couplers on the chip.

Consequently, a scale factor, the *ζ*-coefficient, was applied to the flux measured in a photometric tap before using it to correct the bias in the null depth. This coefficient is defined by

4 $${\zeta }_{i}^{\pm }=\frac{{I}_{i}^{\pm }}{{I}_{p,i}}$$

where ${I}_{i}^{\pm }$ is the flux of beam *i* in the antinull or null output and *I*_*p*,*i*_ is the flux of beam *i* in the photometric output. This coefficient was measured when only one beam is illuminated (Fig. 4c). These *ζ*-coefficients not only took into account the spectral variations of the splitting and coupling ratios, but also the instrumental transmission coefficients between the chip and the detector, ensuring a direct estimation of the contribution of a beam in the coupler over all wavelengths. Following this analysis, it was possible to determine the contribution of any given beam to the null or anti-null signal by using the measurement of flux from the photometric tap.

### Data processing

The data processing relies on the NSC method, which has been adapted to this particular configuration of GLINT that has multiple baselines, and expanded to encompass spectral dispersion^36^. NSC uses measured statistical distributions of the various seeing-induced and instrumental perturbations along with the measured null depth to determine the underlying source null depth. This self-calibration reduces or removes the reliance on observations of calibrator stars. It has been shown that operation over large bandwidths introduces a bias that limits the attainable null depth^17^. In spectrally dispersing the light, GLINT observes narrow bands, minimising any bias and effectively approaching monochromatic performance. Spectrally dispersing light also yields new information, both astrophysical and instrumental. Fluctuations in injection and phase variations are key to the measurement of the source null depth but are affected by chromaticity. Biases for both can be more accurately compensated with wavelength-diverse information. Consequently, spectral dispersion reduces bias on null depth measurements and improves precision over non-dispersed detection, as illustrated with on-sky data in the following sections. Astrophysical exploitation of the spectral diversity of null depths is also possible, but not yet done. Whereas fluctuations of injection were directly measured in each spectral channel and used in the data processing, the version of GLINT discussed here was not able to measure the instantaneous phase of the fringes. Thus, the fluctuations of phases were assumed not to be wavelength-dependent to first order; an approximation that had been tested and validated with the results in the following sections. Recovery of instantaneous fringe phase is planned for the next instrument upgrade, however even just correcting for chromatic injection, spectral dispersion delivers enhanced null depth measurements with less systematic error compared to broadband light.

It was assumed that the variations of OPD were normally distributed in the statistical analysis. Although the true distribution was unknown, this assumption was reasonable as the AO system ensured that phase variations remain smaller than 2*π*, preventing drift or phase wrapping of the fringes during the acquisition. However, various atmospheric issues, uncorrected by the AO, could skew the phase variations away from a normal distribution, such as the island effect^37^ which consists of sharp phase discontinuities across the telescope’s spiders mainly exacerbated by thermal effects when the ground wind speed is low. The variations could be as high as *π* radians, so the null and anti-null outputs were swapped. In order to mitigate the impacts of these long-tails with poor phase control and recover a distribution closer to Gaussian, frames were sorted and those with high phase variation removed by a sigma-clipping filter. This filter operated by keeping frames for which the fluxes in the null (anti-null) output lie within two standard deviations from the smallest (largest) value. Frames falling outside sigma-clipping limits for either null or anti-null outputs were discarded from the analysis.

The data processing consisted of two steps: (i) measuring the photometry, detector noise, the null depths and determining their probability density function (PDF) using histograms after applying the sigma-clipping, and (ii) fitting a model PDF—which is a function of the source null depth—to the observed PDF of the null depth.

**Fig. 5:**
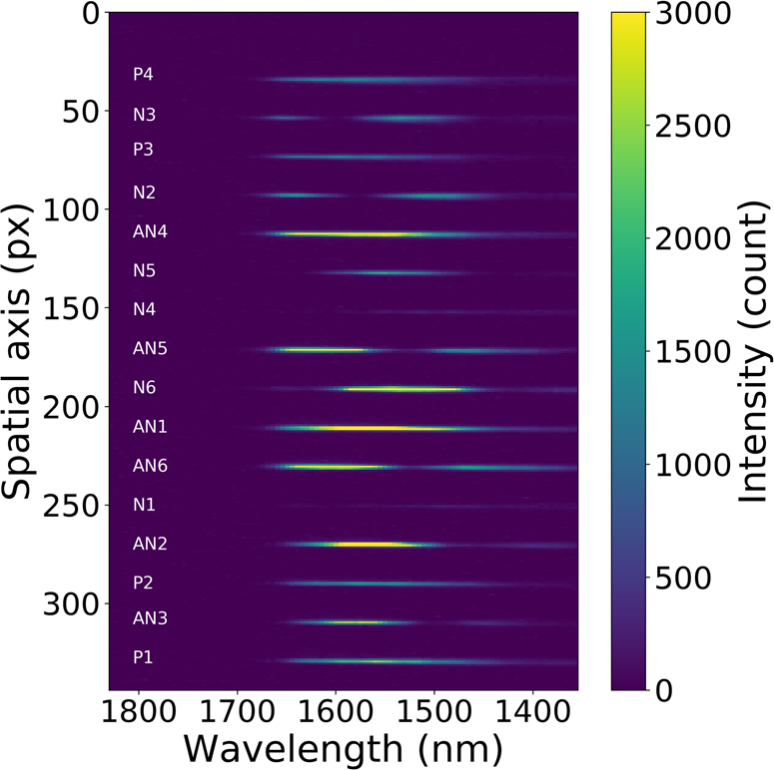
A data frame from the science sensor illuminated with a laboratory source. Spectral dispersion runs along the horizontal direction, while outputs from different waveguides are displaced in vertical rows. The rows labelled “P” are the photometric tap. “N1-6” are the outputs corresponding to the null output and “AN1-6” are the outputs corresponding to the respective anti-null outputs. On this occasion, pistons on the MEMS mirror were configured to produce a nulled signal on N1 and N4.

The flux of a spectral channel (defined by the width of a pixel) at wavelength *λ* was measured by summing the flux along the spatial axis over the width of each output (Fig. 5). This operation was done for every spectral channel of the 16 outputs, for every remaining frame. The same procedure was performed on dark frames.

Next, data was processed for the baseline of interest over the desired spectral bandwidth. For each of its spectral channels at wavelength *λ*, the histograms of the photometric outputs of the data were calculated. The same operation was done for the detector noise in the interferometric outputs of the dark frames.

The null depth, defined as the ratio of the flux of the null output divided by the flux in the anti-null, was calculated for each spectral channel for all remaining data frames. The uncertainty at each point in the null depth histogram was derived from the number of elements in each bin *i* and follows a binomial distribution:

5 $${\sigma }_{{\rm{obs}},\lambda }^{2}=\frac{{f}_{{\rm{obs}},\lambda }(i)(1-{f}_{{\rm{obs}},\lambda }(i))}{{M}_{{\rm{null}},\lambda }},$$

where *σ*_obs,*λ*_(*i*) is the uncertainty of the normalised histogram *f*_obs,*λ*_(*i*) for the *i*-th bin at the wavelength *λ*, and *M*_null,*λ*_ is the number of data frames measured for this wavelength.

The model of the PDF was generated for each spectral channel from the known distributions of the intensities and detector noise, and from the unknown quantities that are the source null depth ${N}_{{\rm{obj}},ij}=\frac{1-{V}_{{\rm{obj}},ij}}{1+{V}_{{\rm{obj}},ij}}$, the position *μ*_OPD_ and the scale factor *σ*_OPD_ of the OPD normal distribution; they constituted the three free parameters to fit (see “Modelling the null depth” in “Methods”). The generation was done with a Monte-Carlo approach because of the presence of large phase errors despite the AO corrections^29^. The PDF models were fitted to the observed PDF on all spectral channels, simultaneously, to obtain the parameters.

### Laboratory performance: null depth of an unresolved source

The aim of this part of the study was to use null depths measured by GLINT to explore the efficacy of spectral dispersion in nulling. GLINT also enables exploration of the stability of the instrumental null depth between different baselines.

**Fig. 6:**
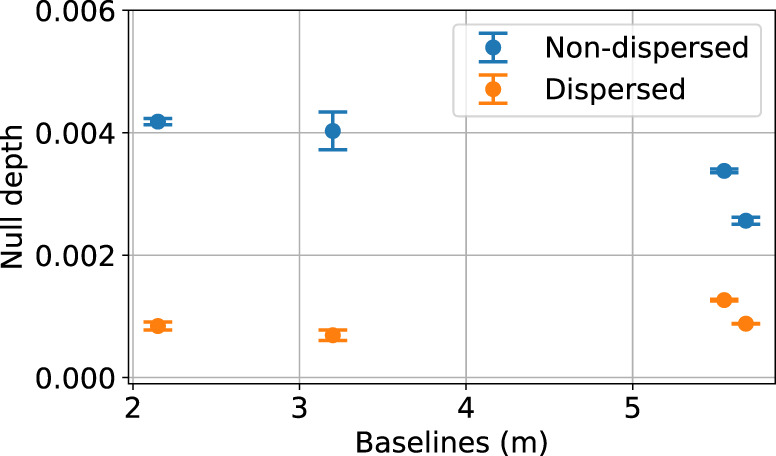
Source null depth with respect to baseline by observing the supercontinuum source of the SCExAO bench. The blue dots represent the null depths measured from the binned spectral channel while the orange dots represent the null depths measured with spectrally dispersed light.

The supercontinuum source of SCExAO was used. It provides a spatially coherent wavefront so that the theoretically-expected null depth should be zero. Atmospheric turbulence was simulated by applying a moving Kolmogorov phase screen to the SCExAO deformable mirror, corresponding to a winds peed of 10 m.s^−1^ with an RMS wavefront error of 200 nm (~*λ*/8). The null depth was then calculated in the bandwidth between 1525 and 1575 nm both with and without the spectrally dispersed signal (the latter obtained by binning the spectral channels). First, the observed null depths appear to be independent of baseline (Fig. 6), confirming the expected stability of the instrument and spatial coherence of the source.

Second, the instrumental non-dispersed null is around 3.5 ± 0.31 × 10^−3^ over a bandwidth of 50 nm. The use of spectral dispersion over the same bandwidth allows us to reach a null depth 3.8 times deeper at 0.92 ± 0.11 × 10^−3^ and with a similar improvement in measurement precision.

This improvement between the dispersed and the non-dispersed null depths and their measurement precision illustrates the influence of the bandwidth on the measurements, and the improvement afforded by the spectrally-dispersed measurement and model. Even so, the null depth is not quite zero, which may be due to factors not included in the model such as blurring of the fringes by vibrations faster than the frame rate.

### Performance on sky: measurement of two stellar diameters

GLINT was deployed on-sky to determine the apparent angular diameters of *α* Bootis and *δ* Virgo.

**Fig. 7:**
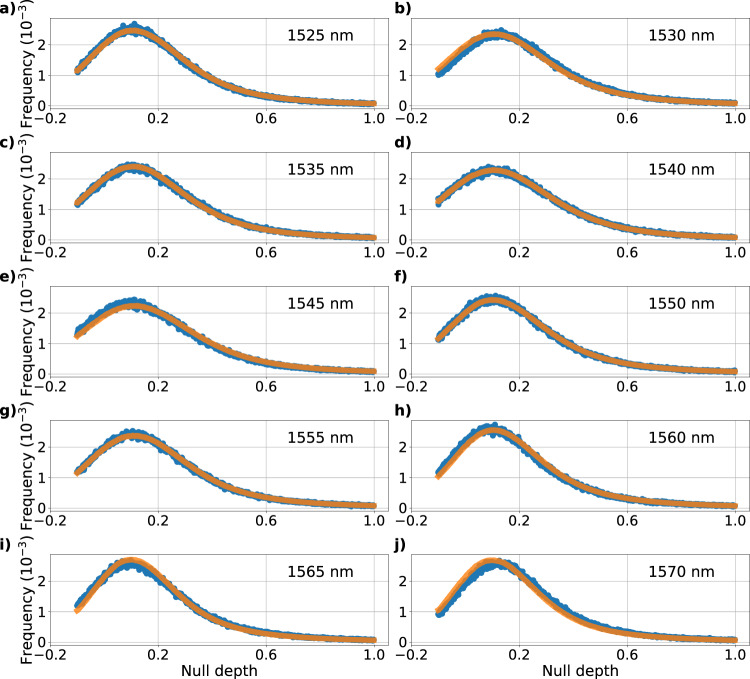
Histograms of the measured null depth of base N1 computed over 10 dispersed spectral channels with the fitted curves. Each subplot from (**a**) to (**j**) shows the histogram of the measured null depth of base N1 on *α* Boo (blue dots) and the fitted model (orange curve), in each of the spectral channel spanning from 1525 to 1570 nm. The fitted parameters are *N*_obj,12_ = 7.05 × 10^−2^ ± 1.86 × 10^−4^, *μ*_OPD_ = 302 ± 0.465 nm and *σ*_OPD_ = 163 ± 0.163 nm for a reduced *χ*^2^ of 2.51.

*α* Bootis is a red giant branch star, with magnitude in H band of −2.81 mag and with published angular diameter measurements between 19.1 and 20.4 mas^38^ in K band. This is around 40% of the *λ*/*D* diffraction limit of the telescope in our band. The average seeing during the night of the 20th of June 2020 ranged between 0.3 and 0.5 arcseconds at 1600 nm, but with some significant wavefront error resulting from low-wind-effect and telescope vibrations. Data were acquired for 15 min at a frame rate of 1400 Hz for the pairs of baselines N1, N4 and N5, N6. The null depth was measured for all baselines on a spectrally dispersed signal from 1525 to 1575 nm. An illustrative example of model fitting to the histograms of the null depth of the baseline N1 on *α* Boo is given in Fig. 7.

*δ* Vir is also a red giant branch star, with a magnitude in H band of −1.05 mag and a measured angular diameter of 10.6 ± 0.736 mas in H band^39^, i.e., around 20% of the diffraction limit. The conditions of observation for the night of the 5th of July 2020, and the configuration of the acquisition were the same as for *α* Bootis, detailed above.

Once the null depths were obtained for the four baselines, the angular diameter of *α* Bootis was deduced from the fitting of these four null depths by a model that gave the expected null leakage given the stellar size, here parameterised by a uniform disk diameter:

6 $${N}_{{\rm{UD}}}=\frac{1-{V}_{{\rm{UD}}}}{1+{V}_{{\rm{UD}}}},$$

so that

7 $${V}_{{\rm{UD}}}=\left|2\frac{{J}_{1}\left(\pi {\theta }_{{\rm{UD}}}\frac{B}{\lambda }\right)}{\pi {\theta }_{{\rm{UD}}}\frac{B}{\lambda }}\right|$$

where *J*_1_ is the Bessel function of the first kind, *θ*_UD_ is the angular diameter, *B* the baseline and *λ* the wavelength.

**Fig. 8:**
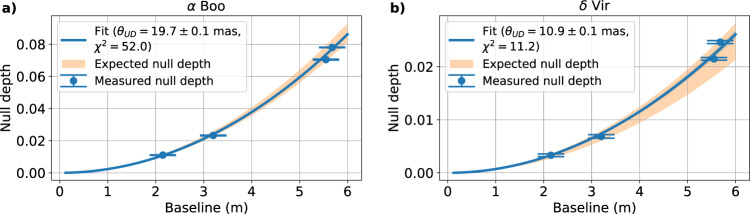
Variations of the source null depth of *α* Boo and *δ* Vir and their respective fitted curves with respect to the baseline. **a** Variation of the source null depth of *α* Boo (blue dots) as a function of baseline together with the best-fit model (blue solid curve). The orange area highlights the expected range of null depth at 1550 nm for an expected UD diameter between 19.1 and 20.4 mas (obtained from the literature). Error bars represent the standard error of the measurements. **b** Source null depth of *δ* Vir (blue dots) with baseline together with the best-fit model (blue solid curve). The orange gives the expected range of null depth for a UD diameter between 9.86 and 11.3 mas (from the literature). The error bars represent the standard deviation of the fitted value of the source null depth given by the covariance matrix of the fit of the histogram, rescaled by the reduced *χ*^2^ of that fit.

The result of this fit of null depth as a function of the baseline is given in Fig. 8a. The four source null depth points are an excellent match to the form of the expected curve and yield parameters within the range of expected literature values. The angular diameter found is 19.7 ± 0.1 mas with a reduced *χ*^2^ of 52.0. The uncertainty has been rescaled by the square root of the reduced *χ*^2^. The *χ*^2^ is high because the error bars are derived only from statistical diversity in the data and do not account for systematics (for example blurring due to seeing variations faster than the frame rate.)

To evaluate the efficacy of spectral dispersion, the stellar diameter was also obtained from spectrally binned null depth data over the same bandwidth. The found diameter is 24.2 ± 1.7 mas, which is systematically larger than the established value (between 19.1 and 20.4 mas). This overestimation of null depth is explained by the fact the fringes of the two baselines N5 and N6 could not be set to the central dark fringe (Fig. 3e) causing a systematic loss of coherence. The spectrally dispersed signal allows this to be accounted for in the model resulting in the delivery of the expected value of null depths.

The histograms of the null depths for *δ* Vir were processed and fitted in the same way. As before, the four source null data points yield an excellent fit to the model (Fig. 8b), which in turn is in agreement with literature expectations for this star (expected UD diameter of 10.6 mas). The angular diameter found is 10.9 ± 0.1 mas with a reduced *χ*^2^ of 11.2 where the uncertainty has been rescaled as before. As for *α* Boo, the high *χ*^2^ betrays the presence of systematic errors not taken into account in the measurement of the null depth, such as high-frequency phase jitter^40^. One option would be to combine the self-calibration with a standard PSF-reference star calibration.

## Discussion

GLINT embodies a new generation of source nulling interferometers. It exploits photonic technology to create multiple interferometer baselines which can be simultaneously nulled. Active optical elements within the instrument allow delays to be finely adjusted, switching different pairs into null operation, or even swapping null and anti-null configuration. The instrument also boasts multi-channel spectral information by way of a spectroscopically dispersed back-end.

Source null depths measured on the laboratory test-bench with a point-like source and a seeing simulator are better than 10^−3^, 3.8 times deeper than that obtained using the non-spectrally-dispersed method. These measurements also gave results consistent with on-sky data, yielding precision of the order of 10^−4^, describing the random statistical error contribution. GLINT was demonstrated on-sky with measurements of the diameters of two stars, both well below the formal *λ*/*D* diffraction limit of the telescope (the smaller of the two being 5 times less than the diffraction limit). The spatial angular scales probed by GLINT are of immediate relevance to direct imaging campaigns targeting exoplanets in the habitable zone around nearby star systems. Considered individually, the raw performance metrics of GLINT are comparable with the best previous-generation nullers, however this instrument shows a pathway in which the straightforward ability to scale photonics technology up to encompass more beams, more combined baselines and incorporate spectral diversity both optically and as an integrated aspect of the data reduction. The goal to span the entire telescope pupil, making use of the full light gathering capability of a large telescope, is within reach.

These ambitions point the way to future improvements in the design of next-generation photonic chips for nulling interferometry. The achromatic performance of the couplers, and the internal and external optical path length matching should both be better controlled to enable straightforward operation of a larger number of simultaneous nulled baselines. This would form a base for the deployment of photonic nulling interferometers on future ELTs or interferometers, such as in the mid-infrared for the VLTI^41^. Further areas of research include management of wavefront errors, and new architectures capable of recovery of the instantaneous baseline phase, while delivering astrophysical data within the same device. Such improvements would deliver feedback to adaptive optics systems tasked with correcting low-order aberrations, with immediate gains in performance for future extremely large telescopes, better data calibration, and improved flagging to reject problematic data that are caused by AO instabilities (for example, the “island effect”).

## Methods

### Specifications of the instrument

The waveguides of the GLINT chip are inscribed in a monolithic block of boroaluminosilicate glass (Schott AF-45) of 75 × 3.5 × 2 mm (length × width × height) by using the Ultrafast Laser Inscription method (ULI hereafter)^42–45^. ULI allows the fast manufacturing of complex single-mode waveguide in three dimensions as well as splitters and codirectional couplers.

The remapper is designed with an S-shape “side-step” to avoid coupling unguided stray light (Fig. 2a)^46^. The output waveguides are translated by 3 mm with respect to the inputs over the first 25 mm while keeping the optical path length of the waveguides matched to within 0.17 μm. In addition, the smallest bend radius is 42 mm and the closest proximity is 60 μm. Keeping within these design constraints results in minimal loss of light at bends in the waveguides and eliminates unwanted coupling effects between them^47^.

Following the remapper, a set of couplers performs the interference between inputs to produce the nulling (Fig. 2b–d). This section of the chip spans a length of 45 mm, a width of 2 mm and a height of 0.08 mm. The first operation in the coupling section is to split each of the four waveguides from the remapper into four individual waveguides.

One of these is routed directly to the output where it forms the photometric tap, yielding a measurement of the flux injected into that waveguide. The remaining three are each paired with the waveguides from the three other inputs via codirectional couplers where the beams interfere by evanescent coupling. The interaction length is tailored so that the coupler behaves as a 50/50 beam splitter at the centre of the H band: the length is 5 mm, and the proximity is 0.01 mm.

The coupling process shifts the phases of the wavefronts from both coupled waveguides by $\frac{\pi }{2}$ during the process^48^. Consequently, a phase-shift of $\frac{\pi }{2}$ should be added to create destructive interference in one waveguide after the coupler and constructive in the other. This is done by pistoning the segmented mirror which induces an air-delay in the optical path; the phase-shift is chromatic so the piston is set to create destructive interference at 1.55 μm.

The GLINT chip provides six interferometric baselines which range from 2.15 to 6.45 m (Table 1) and photometric taps. The photometric, null and antinull outputs are redirected toward the spectrograph, whose spectral resolution is *R* = 160 at 1.55 μm. It consists of an AusOptic 16-channel V-groove glass array with a 127 *μ*m pitch and an angled physical-contact (APC) fibre connector, in which the photonic chip output fibres are set, a collimator with a focal length of 65.1 mm then a BK7-glass 1-inch equilateral prism from Altechna. The sixteen dispersed beams are focused, by a lens of focal length of 150 mm, on the detector.

**Table 1:** Identification table cross-referencing the nulled and anti-nulled outputs with the corresponding pair of beams together with the length of the baseline on sky.

(Anti-)null	Pair of beams	Baseline (m)
(A)N1	1-2	5.55
(A)N2	2-3	6.45
(A)N3	1-4	4.65
(A)N4	3-4	2.15
(A)N5	3-1	3.2
(A)N6	2-4	5.68

The numbering is as adopted in Fig. 1b.

Photodetection is performed with a C-Red2 InGaAs camera, replacing the photodetectors used in the previous version of GLINT. The total detector area is cropped around the dispersed beams so that the dimensions of the grabbed frames are reduced to 344 × 96 pixels (Fig. 5), allowing a maximum frame rate of 1400 Hz, i.e., an integration time of 0.7 ms. This value is around 7 times faster than the median atmospheric coherence time of 5.14 ms at Mauna Kea^49^ so the turbulent phase screen is effectively “frozen” for each frame. The subsequent improvement of the instrumental null is counterbalanced by a reduction in the final sensitivity due to the read-out noise of the detector at high frame rates. Future evolution of the instrument plans to use an e-APD camera with better (less than one electron) read-out noise.

### Observing procedure

Due to the optical path delay limitations noted in the subsection “Characterisation of the photonic chip” in “Results”, simultaneous nulls on four baselines at once were not achievable and so observations were performed by nulling two baselines at a time.

The instrument was aligned using the supercontinuum source integrated into the SCExAO bench the day before the night of observation. The static aberrations on the bench were corrected by the deformable mirror to deliver a flat wavefront, and an active tip-tilt control loop prevented any drift during the alignment procedure of GLINT.

The first step consisted of maximising the injection of the light into GLINT by moving the tip-tilt positions of the segments of the MEMS. The real-time software of GLINT automatically performs this operation by mapping and interpolating the injection with respect to the tip-tilt of the segments.

The second and last step consisted in finding the appropriate delay to be added by the MEMS mirror to achieve the deepest null depth for each baseline. Each baseline was scanned by pistoning the appropriate MEMS segment while the total flux of the null output was monitored, and fitted with a sine function to find the point corresponding to maximum contrast and to determine the actual OPD of the dark fringe for data processing. The optimal set of values for tip/tilt and piston were then stored.

Once on-sky, these optimisation scans were repeated with starlight (in a smaller domain about the optimum found in the lab, to save time). This accounted for any mechanical drifts in the instrument between the lab and on-sky measurements, different static aberrations and atmospheric angular dispersion. The drift in terms of OPD between the lab optimisation and on-sky was found to vary by between *λ*/8 and *λ*/2. Once the pair of baselines was optimised, data was acquired for 15 min with a frame rate of 1400 Hz. The same procedure was repeated for the second pair of baselines. Finally, dark frames were acquired for 15 min by closing the shutter (Fig. 1a).

### Modelling the null depth

Analysis of nulling interferometry data by way of statistical distributions has been described by various authors^24,29^; here the model used is detailed with specific reference to extensions in the method for the richer set of observables delivered by GLINT.

The interference between two beams within the chip is performed in a codirectional coupler, with the relationship between the inputs and the outputs of the coupler written as^48^:

8 $$\left(\begin{array}{l}{a}_{{\rm{out}},i}\\ \,{a}_{{\rm{out}},j}\end{array}\right)=\left(\begin{array}{ll}\cos ({\kappa }_{ij}{L}_{ij})&-j\sin ({\kappa }_{ij}{L}_{ij})\\ -j\sin ({\kappa }_{ij}{L}_{ij})&\cos ({\kappa }_{ij}{L}_{ij})\end{array}\right)\left(\begin{array}{l}{a}_{{\rm{in}},i}\\ {a}_{{\rm{in}},j}\end{array}\right),$$

where *a*_in,*i*/*j*_ (respectively *a*_out,*i*/*j*_) is the complex amplitude of the incoming monochromatic wavefront of modulus ∣*a*_in,*i*/*j*_∣ and phase *ϕ*_*i*/*j*_ (resp. the outcoming monochromatic wavefront of ∣*a*_out,*i*/*j*_∣ and phase ± Δ*ϕ* = ± (*ϕ*_*i*_ − *ϕ*_*j*_)). *κ*_*ij*_ and *L*_*ij*_ are respectively the coupling coefficient depending on the wavelength, and the coupling length of the coupler.

The wavefront injected into the waveguides has a phase offset of $\frac{\pi }{2}$ imposed (Eq. (8)), so that the fluxes at the output of the coupler ∣*a*_out,*i*_∣^2^ and ∣*a*_out,*j*_∣^2^ are respectively described by:

9 $${I}^{-}={I}_{i}\sin {({\kappa }_{ij}{L}_{ij})}^{2}+{I}_{j}\cos {({\kappa }_{ij}{L}_{ij})}^{2}-2\sqrt{{I}_{i}{I}_{j}}\cos ({\kappa }_{ij}{L}_{ij})\sin ({\kappa }_{ij}{L}_{ij})\times {V}_{{\rm{obj}},ij}\sin \left({{\Delta }}\phi \right)+{I}_{{\rm{dark}}}^{-}$$

and

10 $${I}^{+}={I}_{i}\cos {({\kappa }_{ij}{L}_{ij})}^{2}+{I}_{j}\sin {({\kappa }_{ij}{L}_{ij})}^{2}+2\sqrt{{I}_{i}{I}_{j}}\cos ({\kappa }_{ij}{L}_{ij})\sin ({\kappa }_{ij}{L}_{ij})\times {V}_{{\rm{obj}},ij}\sin \left({{\Delta }}\phi \right)+{I}_{{\rm{dark}}}^{+},$$

which are respectively the flux of the destructive and constructive interference, at wavelength *λ*, measured at their respective null and anti-null outputs. *I*_*i*_, *I*_*j*_ are the contribution of the beams *i* and *j* to the interference. *V*_obj,*ij*_ is the modulus of the visibility of the observed object measured at baseline *i*, *j*. ${I}_{{\rm{dark}}}^{\pm }$ is the detector noise in the anti-null or null output, respectively. The term Δ*ϕ* can be explicitly written as

11 $${{\Delta }}\phi =\frac{2\pi }{\lambda }({\delta }_{0,ij}+{{\Delta }}\delta ),$$

where *δ*_0_ is the instrumental OPD between the beams set to the deepest null and Δ*δ* is the variation of OPD caused by the atmospheric turbulence around *δ*_0_.

The intensities *I*_*i*_ and *I*_*j*_ are known thanks to the photometric taps while the *ζ*-coefficients were recovered from test data, so that the Eqs. (9) and (10) are rewritten as follows:

12 $${I}^{-}={I}_{p,i}{\zeta }_{i}^{-}+{I}_{p,j}{\zeta }_{j}^{-}-2\sqrt{{I}_{p,i}{I}_{p,j}}\sqrt{{\zeta }_{i}^{-}{\zeta }_{j}^{-}}\times {V}_{{\rm{obj}},ij}\sin \left(\frac{2\pi }{\lambda }({\delta }_{0}+{{\Delta }}\delta )\right)+{I}_{{\rm{dark}}}^{-}$$

and

13 $${I}^{+}={I}_{p,i}{\zeta }_{i}^{+}+{I}_{p,j}{\zeta }_{j}^{+}+2\sqrt{{I}_{p,i}{I}_{p,j}}\sqrt{{\zeta }_{i}^{+}{\zeta }_{j}^{+}}\times {V}_{{\rm{obj}},ij}\sin \left(\frac{2\pi }{\lambda }({\delta }_{0,ij}+{{\Delta }}\delta )\right)+{I}_{{\rm{dark}}}^{+}.$$

*I*_*p*,*x*_ is the flux of beam *x* = *i*, *j* in the photometric output and ${\zeta }_{x}^{\pm }$ is the ratio of the flux of beam *x* in the anti-null or the null output over the flux of this beam in the photometric one. Finally, the measured null depth is defined by

14 $$N=\frac{{I}^{-}}{{I}^{+}}.$$

The model of the PDF was obtained based on the NSC method and with a Monte-Carlo approach. Synthetic sequences of the intensities *I*_*p*,*i*/*j*_, the detector noises ${I}_{{\rm{dark}}}^{\pm }$ were created from their measured distributions for each spectral channel, and a synthetic sequence of OPDs from a Gaussian distribution of parameters *μ*_OPD_ and *σ*_OPD_. These values were injected into the model (Eqs. (12)–(14)) to create the synthetic sequence of spectrally-dispersed null depths. The joint distributions of null depths between the spectral channels were deduced and compared to the measured joint histograms. It was found that each sequence required around 10^8^ realisations to produce consistent results between Monte-Carlo model evaluations.

The parameter space is periodic with respect to *μ*_OPD_ because of the sine function in the model. Scans of the fringes (see “Observing routine” in “Methods”) were used to determine the approximate *μ*_OPD_ of the measured null. Usually, this is roughly $\frac{\lambda }{4}$ (the measurement was taken at the true, darkest null). But due to the limitations imposed by the OPD mismatches in the chip and the available piston range of the MEMS segments, some measurements were performed in nulls up to 7 fringes away from the central null in non-dispersed light. The loss of coherence was taken into account in the model to deliver null depths corrected for this effect.

Next, the parameter space surrounding the measured OPD was coarsely gridded to find bounds for the subsequent gradient-descent fit. Finally, the non-linear least-square Trust Region Reflective algorithm^50^ was used to find the parameters of the model and particularly the source null depth. The uncertainties of the fitted parameters were deduced from the pseudo-inversion of the Jacobian matrix according to the Moore-Penrose pseudo inversion method then they were rescaled by the reduced *χ*^2^ of the fit.

## Data Availability

The data produced in this study are available from the corresponding author on reasonable request.

## Code availability

The code of the data reduction of GLINT is available on Github^36^: https://github.com/SydneyAstrophotonicInstrumentationLab/GLINTPipeline. The documentation is hosted on the platform Readthedoc.org: https://glintpipeline.readthedocs.io/en/latest/.
